# MiRNA Omics Reveal the Mechanisms of the Dual Effects of Selenium Supplementation on the Development of the Silkworm (*Bombyx mori*)

**DOI:** 10.3390/ijms26073394

**Published:** 2025-04-04

**Authors:** Wen-Jie Ge, Fei Hu, Ting-Ting Hong, Kiran Thakur, Shun-Ming Tang, Jian-Guo Zhang, Zhao-Jun Wei

**Affiliations:** 1 School of Food and Biological Engineering, Hefei University of Technology, Hefei 230601, China; 2022111350@mail.hfut.edu.cn (W.-J.G.); hufei@hfut.edu.cn (F.H.); 2022111342@mail.hfut.edu.cn (T.-T.H.);; 2 School of Biological Science and Engineering, North Minzu University, Yinchuan 750021, China; 3 Jiangsu Key Laboratory of Sericultural Biology and Biotechnology, School of Biotechnology, Jiangsu University of Science and Technology, Zhenjiang 212003, China; 4 Key Laboratory of Silkworm and Mulberry Genetic Improvement, Ministry of Agriculture, Sericultural Research Institute, Chinese Academy of Agricultural Sciences, Zhenjiang 212018, China

**Keywords:** *Bombyx mori*, miRNA, Se, gene regulation networks

## Abstract

This study explores the dual effects of selenium (Se) supplementation on silkworm development by analyzing miRNA expression profiles of fat bodies in silkworms under different Se concentrations (50 µM and 200 µM). Compared to the control, 84 miRNAs displayed different expression levels in the F_50 µM group, with 72 miRNAs up-regulated and 12 down-regulated; 152 miRNAs were differentially expressed in the F_200 µM group, with 124 up-regulated and 28 down-regulated. In the F_50 µM group, the target genes of differentially expressed miRNAs were mainly enriched in Toll and Imd signaling pathways, oxidative phosphorylation, and ribosome biogenesis in eukaryotes; however, mainly oxidative phosphorylation, ribosome biogenesis in eukaryotes, and the spliceosome were enriched in the F_200 µM group. Based on the results of the protein–protein interaction network and miRNA-target network, bmo-miR-2a-1-5p and bmo-miR-317-3p_L-2R+2 were screened as key miRNAs in the F_50 µM group and the F_200 µM group, respectively. The bmo-miR-2a-1-5p mainly targeted 10014128 (*DREDD*), 100862750 (*ATF2*), and 101744000 (*Tak1*) genes, which were enriched in Toll and Imd signaling pathways. The bmo-miR-317-3p_L-2R+2 primarily regulated 101738508 (*SF3b*) and 101746688 (*Prp19*) genes, which were in the spliceosome pathway. Thus, our results demonstrated that Se supplementation improved the silkworm development via bmo-miR-2a-1-5p miRNA regulation of the Toll and Imd signaling pathways and inhibited it via bmo-miR-317-3p_L-2R+2 miRNA targeting the spliceosome pathway. Our data revealed that 50 µM Se supplementation could improve silkworm productivity; meanwhile, a 200 µM Se treatment displayed toxic effects, leading to impaired development.

## 1. Introduction

Selenium (Se), a vital trace element for human health, has broad applications, spanning from basic biology to clinical medicine [1]. Due to its anti-inflammatory, anti-cancer, and antioxidant qualities, Se has been used to treat Leukemia, AIDS, cardiovascular disease, and other illnesses [2,3]. In agriculture, Se can be applied as a fertilizer to increase the soil’s Se concentration, thereby enhancing crop quality and yield [4,5]. Agricultural insects supplemented with Se have shown improved immunity, growth rate, survival, and reproductive efficiency [6,7]. For instance, *Tenebrio molitor* exhibited rapid development and decreased larval mortality when exposed to specific Se concentrations while *Drosophila melanogaster* showed enhanced survival and egg production at moderate levels of Se [8,9].

Silkworms (*Bombyx mori*) serves as a typical research model in biology, genetics, and entomology [10]. Their growth, development, and reproductive function are affected by various environmental factors, including temperature, humidity, nutrients, and micronutrients [11,12]. Our previous studies revealed the dual effects of Se supplementation on silkworm development [13]. Specifically, 50 µM of Se supplementation promotes silkworm larval growth and improves cocoon quality and reproduction, whereas 200 µM of Se exhibits toxicity on silkworms by inhibiting their growth and development [14]. In addition, there were significant differences in the ability of different tissues of the silkworm to accumulate Se, which could be related to the mechanisms of Se distribution and metabolism in the organism [14]. Further studies have also shown that Se has an important effect on gene expression, as well as antioxidant and detoxification systems in the fat body of the silkworm [15]. As a crucial metabolic organ, the fat body is involved in the regulation of various physiological functions. However, scant information is available for miRNA expression in the silkworm fat body at different concentrations of sodium selenite.

MicroRNAs (miRNAs) are small endogenous RNAs, approximately 22 nt in length, that play a crucial role in regulating gene expression at the post-transcriptional stage [16]. The processes involved in the biogenesis, target recognition, and degradation of these precisely regulated miRNAs indicate that maintaining miRNA homeostasis is essential for regulating cellular processes, such as proliferation, growth, differentiation, and apoptosis. The ectopic overexpression of bmo-miR-71 and bmo-miR-252 in *BmN* cells led to a significant down-regulation of *Seroin2* expression at the transcriptional level, which, in turn, diminished the infectivity of *BmNPV* [17]. Bmo-miR-3351 regulates glutathione content and inhibits *BmNPV* proliferation by targeting *BmGSTe6* in *Bombyx mori* [18]. Based on these findings, our study aimed to explore how miRNAs may contribute to the understanding of the dual effects of selenium on silkworms.

To investigate the mechanism behind the dual effects of Se supplementation on silkworm growth and development, we previously reported the metabonomic analysis of the hemolymph [19] and transcriptome analysis of the fat body [15]. As a continuation of this research, we analyzed the miRNA expression profiles of the fat body in silkworms supplemented with 50 µM of Se (definition as F_50 µM group) and 200 µM of Se (definition as F_200 µM group). The findings from this silkworm-based ecotoxicological study provide a valuable framework for understanding nutrient-dependent developmental plasticity to boost the economic value of insects and support sustainable growth in the insect farming sector, as well as potentially leveraging its possible health advantages.

## 2. Results

### 2.1. Analysis of Differentially Expressed miRNAs

**Figure 1:**
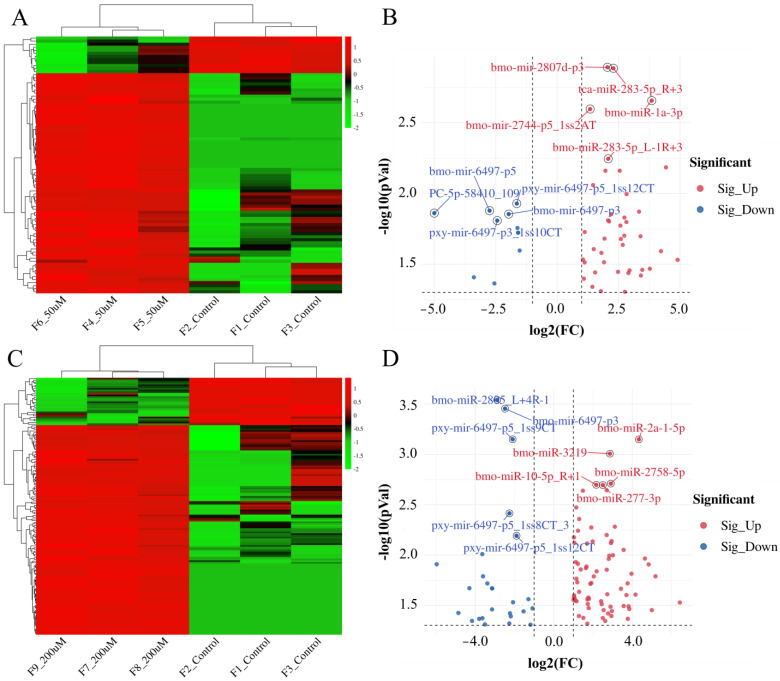
The identified miRNAs in the fat bodies of silkworms. (**A**) the heat map between F_50 µM group and the control. (**B**) the differentially expressed miRNA between the F_50 µM group and the control. (**C**) the heat map between the F_200 µM group and the control. (**D**) the differentially expressed miRNA between the F_200 µM group and the control.

The mature microRNAs and their precursors were analyzed using the miRBase database (http://www.mirbase.org/, accessed on 10 July 2024) and the numbers were identified as 295, 402, and 429 from the control group, F1_50 μM group, and F1_200 μM group, respectively (Table S1). Following Se treatment, there was a significant alteration in the expression of differentially expressed miRNAs within the fat body of the silkworm, exhibiting a high degree of interrelatedness (Figure 1A,C). The volcano plot revealed that 84 differentially expressed miRNAs were identified in the fat body of the F_50 µM group, of which 72 miRNAs were up-regulated and 12 were down-regulated. Among these, bmo-mir-2807d-p3, tca-miR-283-5p_R+3, bmo-miR-1a-3p, bmo-mir-2744-p5_1ss2AT, and bmo-mir-2818-2-p3 demonstrated significant up-regulation while pxy-mir-6497-p5_1ss12CT, bmo-mir-6497-p5, PC-5p-58410_109, bmo-mir-6497-p3, and pxy-mir-6497-p3_1ss10CT exhibited significant down-regulation (Figure 1B). In the F_200 µM group, a total of 152 differentially expressed miRNAs were identified, with 124 showing up-regulation and 28 showing down-regulation. Notably, bmo-miR-2a-1-5p, bmo-miR-3219, bmo-miR-2758-5p, bmo-miR-10-5p_R+1, and bmo-miR-277-3p were significantly up-regulated, whereas bmo-miR-2805_L+4R-1, bmo-mir-6497-p3, pxy-mir-6497-p5_1ss9CT, pxy-mir-6497-p5_1ss8CT_3, and pxy-mir-6497-p5_1ss12CT exhibited significant down-regulation (Figure 1D).

### 2.2. Validation of DEMs by RT-qPCR

**Figure 2:**
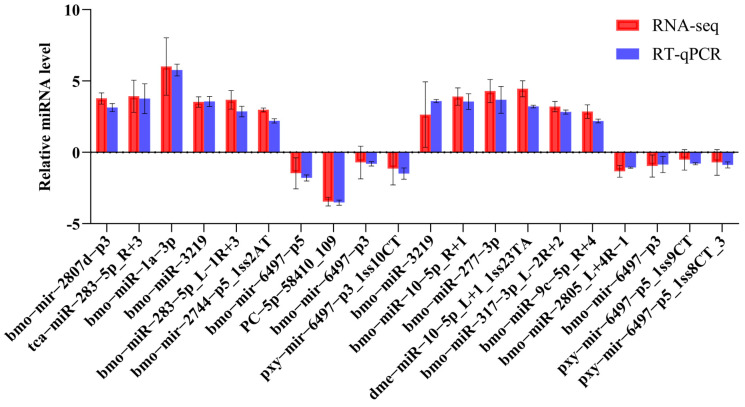
The validation of differentially expressed microRNAs (DE-miRNAs) was conducted using a reverse transcription quantitative polymerase chain reaction (RT-qPCR). The bars represent the relative expression levels of the microRNAs, with all values subjected to log2 transformation.

The expression level of 19 miRNAs was evaluated by a RT-qPCR to validate the authenticity and reliability of the results using miRNA sequencing data (Figure 2). Among the eight miRNAs selected from the fat body of the F_50 µM group, five exhibited an increase in expression while three demonstrated a decrease. In the case of the F_200 µM group, eleven miRNAs were selected, with seven showing an increase in expression and four showing a decrease. For instance, the expression level scores for bmo-miR-2758-5p, as determined by RT-qPCR and RNA sequencing, were 4.62 and 4.87, respectively. These results indicate the potential for a more in-depth investigation of the data generated through miRNA sequencing.

### 2.3. Prediction of Targets of Differentially Expressed miRNAs

Target gene prediction for significantly differentially expressed miRNAs (DE-miRNA) was conducted utilizing the TargetScan (https://www.targetscan.org/vert_80/, accessed on 10 July 2024) and miRanda software platforms (https://www.miranda.software/?lang=zh, accessed on 10 July 2024). In the TargetScan algorithm, target genes with a context score percentile below 50 were excluded while, in the miRanda algorithm, those with a maximum energy value exceeding −10 were eliminated. The final target genes for the differential miRNAs were determined by identifying the intersection of results from both software tools. Specifically, the overlap between the TargetScan and miRanda algorithms yielded 14,680 target genes in the F_50 µM group, whereas the overlap in the F_200 µM group comprised 15,275 target genes (Figure S1).

### 2.4. GO Enrichment Analysis of Target Genes and GO Networks

**Figure 3:**
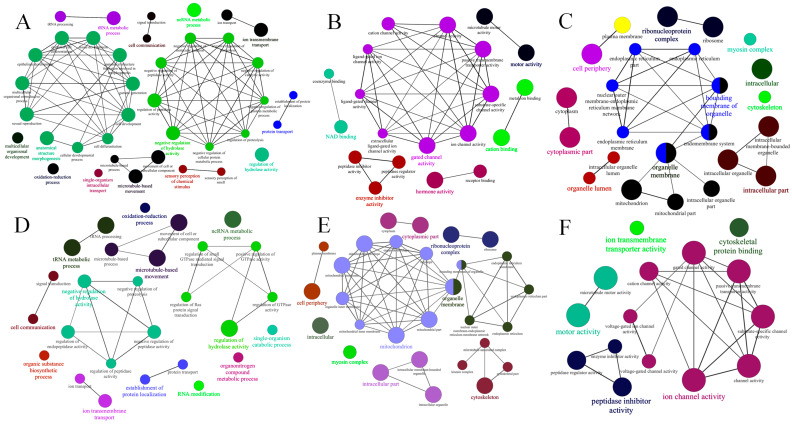
GO-act network of targets. Each set is categorized by the most important term. The size of the nodes indicates the importance of the enrichment. Some related groups partially overlap. (**A**) BP—associated categories in the F_50 μM group; (**B**) MF—associated categories in the F_50 μM group; (**C**) CC—associated categories in the F_50 μM group; (**D**) BP—associated categories in the F_200 μM group; (**E**) MF—associated categories in the F_200 μM group; (**F**) CC—associated categories in the F_200 μM group.

The primary functions of the target genes were predicted through Gene Ontology (GO) analysis (Table S2). In the F_50 μM group (Figure 3A), the most significant GO terms within the biological processes (BPs) category included tRNA metabolic processes (GO:0006399) with a *p*-value of 1.22456 × 10^−18^; ncRNA metabolic processes (GO:0034660) with a *p*-value of 1.78589 × 10^−12^; anatomical structure morphogenesis (GO:0009653) with a *p*-value of 6.6690 × 10^−9^; anatomical structure formation (GO:0048646) with a *p*-value of 1.2153 × 10^−8^; and cell development (GO:0048468) with a *p*-value of 2.7808 × 10^−8^, all of which are implicated in morphogenetic processes (Figure 3A).

In the molecular function (MF) category, the most significant GO terms included molecular activity (GO:0003774) with a *p*-value of 4.1918 × 10^−10^ (Figure 3B); gated channel activity (GO:0022836) with a *p*-value of 8.4085 × 10^−9^; nucleic acid binding (GO:0003676) with a *p*-value of 1.0116 × 10^−8^; substrate-specific channel activity (GO:0022838) with a *p*-value of 2.9183 × 10^−8^; and passive transmembrane transporter activity (GO:0022803) with a *p*-value of 2.9183 × 10^−8^.

Regarding the cellular component (CC) category (Figure 3C), significant enrichment was identified in the cytoplasmic fraction (GO:0044444) with a *p*-value of 6.3699 × 10^−18^, cytoplasmic (GO:0005737) with a *p*-value of 1.1725 × 10^−15^, ribonucleoprotein complex (GO:0030529) with a *p*-value of 3.8518 × 10^−12^, intracellular (GO. 0005622) with a *p*-value of 7.9423 × 10^−9^, and ribosomes (GO:0005840) with a *p*-value of 2.6266 × 10^−8^.

In the F_200 μM group (Table S2), the most significant GO terms in the biological processes (BP)-related category were similarly enriched in the tRNA metabolic process and ncRNA metabolic process (Figure 3D), along with microtubule-based movement (GO:0007018) with a *p*-value of 1.1900 × 10^−9^; movement of cell or subcellular component (GO:0006928) with a *p*-value of 3.3000 × 10^−9^; and regulation of hydrolase activity (GO:0051336) with a *p*-value of 3.8600 × 10^−7^.

In the molecular function (MF) category, significant GO terms included motor activity (GO:0003774) with a *p*-value of 3.5100 × 10^−13^ (Figure 3E); microtubule motor activity (GO:0003777) with a *p*-value of 5.0400 × 10^−10^; substrate-specific channel activity (GO:0022838) with a *p*-value of 1.8800 × 10^−8^; passive transmembrane transporter activity (GO:0022803) with a *p*-value of 1.8800 × 10^−8^; and channel activity (GO:0015267) with a *p*-value of 1.8800 × 10^−8^.

In the GO category related to the cellular component (CC) category (Figure 3F), significant enrichment was observed in the cytoplasmic part (GO:0044444) and *p* = 9.0600 × 10^−21^; cytoplasm (GO:0005737) with a *p*-value of 1.1725 ×10^−15^; ribonucleoprotein complex (GO. 0030529) with a *p*-value of 2.1700 × 10^−16^; ribosome (GO:0005840) and *p* = 4.0900 × 10^−15^; and mitochondrion (GO:0005739) with a *p*-value of 2.0500 × 10^−9^.

### 2.5. KEGG Pathway Enrichment Analysis

**Figure 4:**
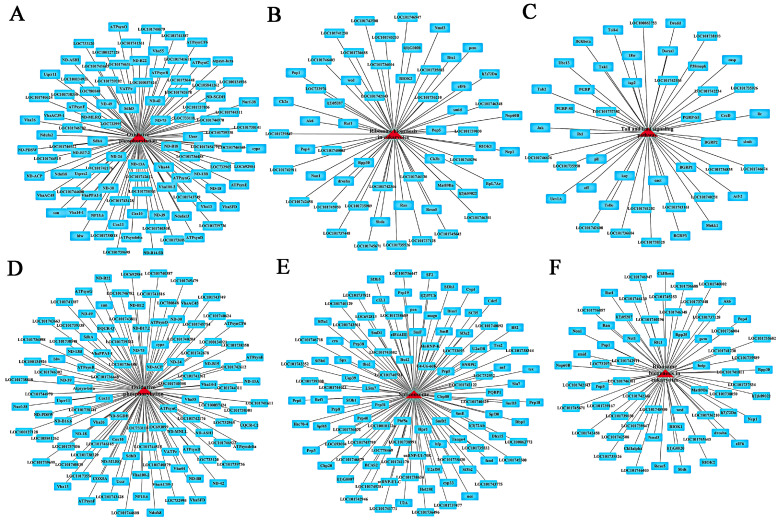
KEGG pathway diagram. Red represents the KEGG pathway and blue represents the enriched targets. (**A**) Oxidative phosphorylation in the F_50 μM group; (**B**) ribosome biogenesis in eukaryotes in the F_50 μM group; (**C**) Toll and Imd signaling pathway in the F_50 μM group; (**D**) oxidative phosphorylation in the F_200 μM group; (**E**) ribosome biogenesis in eukaryotes in the F_200 μM group; (**F**) spliceosome in the F_200 μM group.

The KEGG pathways based on the target genes from the F_50 μM and F_200 μM groups are presented in Table S3. In the F_50 μM group, the most prominent pathway identified was the Toll and Imd signaling pathway (KEGG:04624), which exhibited a significance level of *p* = 6.0716 × 10^−6^, with 46 enriched targets (Figure 4C). Additionally, the oxidative phosphorylation pathway (KEGG:00190) was associated with 98 enriched targets and a significance level of *p* = 2.2650 × 10^−5^ (Figure 4A). The pathway related to ribosome biogenesis in eukaryotes (KEGG:03008) contained 57 targets (Figure 4B), with a significance of *p* = 2.4875 × 10^−4^. In the F_200 μM group, the oxidative phosphorylation pathway (KEGG:00190) emerged as the most significant, encompassing 111 targets and a *p*-value of 1.4600 × 10^−4^ (Figure 4D). Furthermore, the eukaryotic ribosome biogenesis pathway (KEGG:03008) included 64 targets (Figure 4F), with a *p*-value of 2.7358 × 10^−4^ while the spliceosome pathway (KEGG:03040) comprised 108 targets (Figure 4E), with a significance level of *p* = 3.1303 × 10^−4^. The findings indicate a notable distinction between the F_50 μM and F_200 μM groups, particularly concerning the Toll and Imd signaling pathway and the spliceosome pathway, thereby highlighting potential avenues for further investigation (Figure 4B).

### 2.6. PPI Network Establishment and Hub Gene Analysis of Target Genes

**Figure 5:**
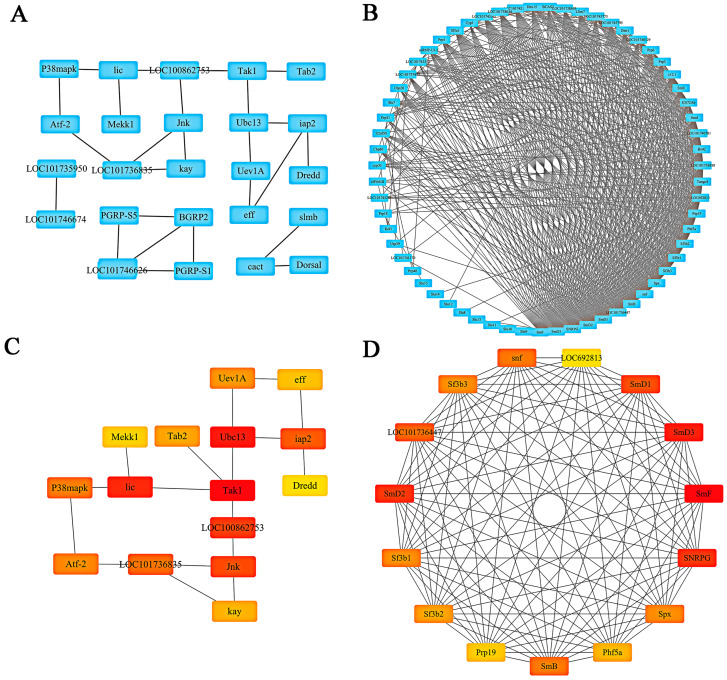
The protein–protein interaction (PPI) network has been enriched with target genes associated with the KEGG pathway. Panel (**A**) illustrates the interactions among annotations for targets within the F_50 μM group in the PPI network. Panel (**B**) depicts the interactions among annotations for targets in the F_200 μM group within the same network. Panel (**C**) presents the network comprising the top 15 scoring targets in the F_50 μM group while Panel (**D**) displays the network formed by the top 15 scoring targets in the F_200 μM group. In these visual representations, the color red indicates higher scores, whereas yellow signifies lower scores.

The protein–protein interaction (PPI) networks of different groups were constructed, which comprised 25 nodes and 26 edges in the F_50 μM group and 55 nodes (Figure 5A) and 661 edges in the F_200 μM group (Figure 5B), respectively. Through an analysis of the network topology, we employed the Cytohubba algorithm to identify the top 15 nodes in each group, thereby determining 15 hub genes for each respective group (Figure 5C,D). The hub genes identified in the F_50 μM group included *Tak1*, *Dredd*, *iap2*, *Uev1A*, *Mekk1*, *Ubc13*, *lic*, *Jnk*, *LOC100862753*, *eff*, *Atf-2*, *Tab2*, *LOC101736835*, *P38mapkz*, and *kay*. Conversely, the F_200 μM group comprised the following hub genes: *SmB*, *snf*, *Sf3b2*, *LOC101736447*, *Phf5a*, *LOC692813*, *SmD3*, *SmD1*, *Sf3b1*, *SNRPG*, *Prp19*, *Sf3b3*, *SmD2*, *SmF*, and *Spx*, as detailed in Table S4.

### 2.7. Construction of the miRNA-Target Regulatory Network

**Figure 6:**
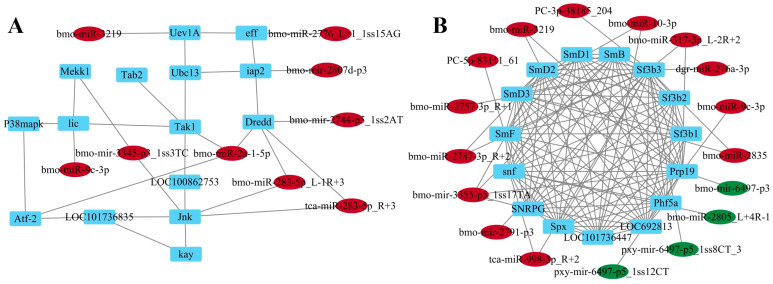
miRNA-target interaction network. (**A**) The network of miRNAs and their targets in the Toll and Imd signaling pathway of the F_50 μM group; (**B**) the network of miRNAs and their targets in the spliceosome pathway of the F_200 μM group. Red color indicates significantly up-regulated miRNAs (*p* < 0.01), green color indicates significantly down-regulated miRNAs (*p* < 0.01), and blue color indicates targets.

The miRNA-target regulatory network was established to identify the significant miRNAs with a *p* < 0.01 level. As illustrated in Figure 6A, the F_50 μM group exhibited nine significantly up-regulated miRNAs and fifteen associated targets. Conversely, Figure 6B indicates that the F_200 μM group comprised four significantly down-regulated miRNAs and thirteen significantly up-regulated miRNAs and regulated a total of fifteen targets. The pivotal miRNA identified in the F_50 μM group, based on network topology analysis, was bmo-miR-2a-1-5p while the key miRNA in the F_200 μM group was bmo-miR-317-3p_L-2R+2, with both miRNAs demonstrating up-regulation.

### 2.8. Construction of the KEGG-miRNA-Targeting Network

**Figure 7:**
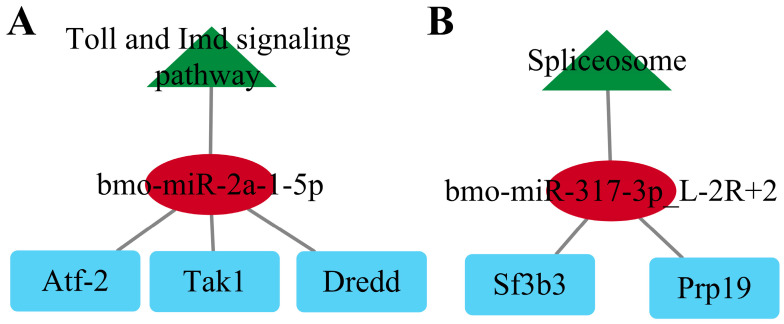
Construction of the KEGG-miRNA-targeting network. (**A**) Representative Toll and Imd signaling pathway regulatory networks in the F_50 μM group. (**B**) Representative spliceosome pathway regulatory network of the F_200 μM group. Triangles represent KEGG pathways, circles represent key miRNAs, and rectangles represent key targets. Red indicates obvious miRNAs. green indicates KEGG pathway. Blue indicates target gene.

To further clarify the roles of these miRNAs, we developed a network that includes target genes, KEGG pathways, and the miRNAs. As illustrated in Figure 7, we established the regulatory network for the F_50 μM group in relation to the Toll and Imd signaling pathways and for the F_200 μM group concerning the spliceosome pathway. The bmo-miR-2a-1-5p is a significant microRNA involved in the Toll and Imd signaling pathways, exhibiting increased expression levels. Its primary targets include *Dredd*, *Atf-2*, and *Tak1* (Figure 7A). Similarly, bmo-miR-317-3p_L-2R+2 plays a crucial role in the spliceosome pathway, also demonstrating up-regulated expression, with its main targets being *Prp19* and *Sf3b3* (Figure 7B).

## 3. Discussion

Our previous research studies have reported that a concentration of 50 µM of sodium selenite can improve the vitality of silkworms [19] while 200 µM of sodium selenite is detrimental to their growth and development [20]. The underlying mechanisms of sodium selenite’s effects on silkworm growth and development remain unclear. Our current study presents comprehensive data on the dual effects of Se supplementation on silkworm development, focusing on miRNA expression profiles and their regulatory mechanisms.

Based on the miRNA omics data, we identified 84 and 152 differentially expressed miRNAs in the F_50 μM group and F_200 μM group, respectively. After 50 μM of Se supplementation, bmo-miR-1a-3p, bmo-mir-2807d-p3, and tca-miR-283-5p_R+3 showed significant up-regulation. Previous research on Se effects in insects indicated that the majority of differentially expressed miRNAs exhibit expression patterns consistent with those observed in our study [21,22,23]. For instance, bmo-miR-1a-3p potentially down-regulates *BmVMP23* expression by binding to its 3′ (UTR), adversely affecting silkworm egg development [24]. In our study, the up-regulation of bmo-miR-1a-3p expression in the F-50 group may inhibit the complementary interaction with the target site, benefiting silkworm development. Additionally, bmo-mir-2807d-p3 regulates multiple components of hormone signaling pathways or proteins associated with hormone biosynthesis in silkworm development [25] while tca-miR-283-5p_R+3 relates to *Tribolium castaneum* [26]. Bmo-mir-6497-p3 regulates the silkworm biological clock during embryonic retention, mainly being involved in red flour beetle development [27]. Its down-regulation in the F-50 group up-regulates genes involved in transport and catabolism, which may involve *GSTe2*, *LOC101740774*, *LOC101745817*, *LOC101746537*, and *LOC101744094*, among others, and the end result is a faster metabolic rate and faster embryo development. Pxy-mir-6497-p3_1ss10CT from *Plutella xylostella* may influence the larval-pupal transition [28], promoting embryogenesis. The down-regulation of PC-5p-58410_109 may inversely activate pathways of metabolic detoxification in the silkworm [29]. Bmo-miR-3219 is expressed in *BmN* cells while Bmo-miR-2758-5p targets BmFMBP-1 and inhibits its expression in *BmN* cells [30]. The up-regulated bmo-miR-10-5p_R+1 inhibits viral replication and enhances disease resistance in *B*. *mori* [31]. Additionally, bmo-miR-277-3p was up-regulated in infected *B.* P50 larvae and this up-regulation is believed to interact with Dnmt2, potentially influencing multiple immune pathways. Bmo-miR-277-3p and bmo-miR-277-5p target Dnmt2 in infected larvae [32], with the gene cluster implicated in development [33]. Bmo-miR-2805 enhances *B. mori* mitogen light chain gene expression [34]. Pxy-miR-6497 may regulate *PxGST*s1’s 3′UTR [35].

In the F_50 μM group, we identified 114,680 target genes, involved in molecular activity, gated channel activity, nucleic acid binding, and substrate-specific channel activity. These target genes were enriched in Toll and Imd signaling pathways, oxidative phosphorylation, and ribosome biogenesis. Notably, the Toll and Imd signaling pathways garnered particular interest due to their critical role in insect innate immunity, representing the most significant pathways enriched by the identified genes [36]. Various micronutrients enhance insect pathogen resistance via the Toll and Imd signaling pathways. Furthermore, cadmium exposure has been shown to diminish both cellular and humoral immunity in *Drosophila*, via the same pathways [37].

This study identified 46 genes enriched in Toll and Imd signaling pathways, including *PGRP-S1 (692372)*, *PGRP-S5 (692398)*, *PGRP-SA (101735926)*, *PGRP-SC*, and *PGRP-LC*, which recognize bacterial peptidoglycan. In the silkworm, twelve *PGRP* genes have been characterized, comprising six long and six short variants [38]. The PGRP-LC membrane does not require the Imd pathway interaction [39]; however, mutations impair Imd pathway activation and resistance to Gram-negative bacteria [40]. Dredd links the Imd pathway to death-associated ced-3/Nedd2-like proteins, with its absence increasing tick bacterial susceptibility [41]. The hormone 20-hydroxyecdysone (ecdysone) directs larval intestinal epithelium destruction via programmed cell death, involving the activation of caspases and the regulation of IAP2 [42]. The BGRP1 (692379) and BGRP2 (693016) proteins, classified as β-1,3-glucan recognition proteins in *B. mori*, trigger innate immune responses against fungal pathogens [43]. Notably, key Toll pathway components, such as *SPz (101741202)*, *MyD88 (101735950)*, *Tube (101746674)*, and *Pelle (101745667)*, were regulated within Group 50. Specifically, the miRNA bmo-mir-6497-p3, which modulates *SPz (101741202)*, exhibited down-regulation. Similarly, the miRNA bmo-mir-6497-p5, which regulates *Pelle (101745667)*, also demonstrated down-regulation, alongside several miRNAs that influence *MyD88 (101735950)* and *Tube (101746674)*, which showed a trend of down-regulation.

In the F_200 μM group, 15,275 target genes were analyzed, primarily related to motor activity, microtubule motor activity, and channel activity. Pathway analysis revealed links to oxidative phosphorylation, ribosome biogenesis, and the spliceosome pathway, the latter particularly enriched with 108 target genes. Among these, *Sm*, *U1-70K*, and *U1C* are components of U1 snRNA [44] while *FBP11*, *S164*, *p68*, *CA150*, and *FUS* are associated with it. *Sm*, *U2A*, *SF3a*, and *SF3b* are part of U2 snRNA, with *U2AF*, *PUF60*, *SPF30*, *CHERP*, *SR140*, and *Prp43* also linked to U2 snRNA [45]. Additionally, *Lsm*, *Sm*, *Prp3*, *Prp4*, *CypH*, and *Prp31* are involved in the formation of U4/U6 snRNA [46]. The tri-snRNA U1 consists of *Sm*, *Snu114*, *Bn2*, *Prp6*, *Prp8*, *Prp8BP*, *Prp28*, and *DIB1* while *Sad1*, *Snu66*, *Sun23*, and *Prp38* are associated with both U4/U6 snRNA and tri-snRNA U1 [47].

Protein–protein interaction (PPI) network analysis in the F_50 μM group identified 15 hub genes, including *Tak1 (101744000)*, *JNKK (100862753)*, *JNk (100126162)*, *Jra (101736835)*, *ATF2 (100862750)*, *P38 (692545)*, *MKK3 (101744017)*, *MEKK1 (101743223)*, *Ubc13 (732928)*, *Uev1A (101743376)*, *Tab2 (101743924)*, *Iap (100529214)*, *Effete (101738264)*, and *Dredd (100141428)*. A miRNA-target regulatory network identified two differentially expressed miRNAs, bmo-miR-2a-1-5p and bmo-miR-317-3p_L-2R+2, in the F_50 μM and F_200 μM groups, respectively, both exhibiting up-regulation. The target of bmo-miR-2a-1-5p is known to regulate three significant targets: *Tak1 (101744000)*, *ATF2 (100862750)*, and *Dredd (100141428)* [48]. For instance, miR-2a-3p affects chitin biosynthesis in the hemipteran *Nilaparvata lugens* and is linked to cold tolerance in *Dermacentor silvarum* (Acari: Ixodidae) [49]. Moreover, Tak1 has been demonstrated to restore immune homeostasis following inhibition [50]. In the context of the F_200 μM group, bmo-miR-317-3p_L-2R+2 up-regulated targets of two highly relevant genes, *SF3b (101738508)* and *Prp19 (101746688)*. Previous studies have indicated that miR-317 affects insect reproduction, larval ovary morphology, and innate immune responses [51], with higher expression being lethal in *B. mori*, suggesting negative regulation of the Imd pathway in this species. Additionally, miR-317 has been shown to negatively influence *Bactrocera dorsalis* feathering [52].

We integrated the KEGG pathway with key miRNAs and their corresponding targets to construct a KEGG-miRNA-target network. In total, 50 µM of selenium (Se) up-regulated bmo-miR-2a-1-5p expression in the fat body, reducing target gene expression. *Tak1* and *Tab2* form a signaling complex within the Imd pathway [53], with disruption triggering a constitutive activation in *Drosophila* [54]. The transcription factor atf2 sustains energetic homeostasis with p38 in the fat body of *Drosophila* [55]. Dredd, a ced-3/Nedd2-like cysteine protease, regulates the apoptosis of *B*. cells [56] and operates directly downstream of the cleavage and activation of Relish [57]. Inhibiting Dredd expression protects silkworm cells against apoptosis, which is critical for the growth and development of the silkworm. Additionally, the miRNA bmo-miR-317-3p_L-2R+2 was found to be up-regulated in the fat body of *B. mori* following treatment with 200 µM Se. This up-regulation negatively regulates the target genes *SF3b* and *Prp9*, leading to destabilization of the spliceosome pathway in silkworms. This causes mRNA degradation via nonsense-mediated decay [58], down-regulating mRNA and protein levels, disrupting gene expression, and potentially impairing cellular functions. The spliceosome pathway’s role in insect immunity suggests suppressed *Prp9* expression may compromise immune responses [59]. In summary, our results demonstrated that Se supplementation improved the silkworm development via bmo-miR-2a-1-5p miRNA regulation of the Toll and Imd signaling pathways and played an inhibitory role via bmo-miR-317-3p_L-2R+2 miRNA targeting the spliceosome pathway. To summarize, our study elucidates the dual role of Se in silkworm development through miRNA-mediated regulation of immune and metabolic pathways. Low Se enhances growth via Toll/Imd activation while high Se inhibits development by disrupting RNA splicing.

## 4. Materials and Methods

### 4.1. Experimental Material

The silkworm strain P50 was used in the present research; it was maintained at the Sericulture Research Institute, Chinese Academy of Agricultural Sciences (Zhenjiang, China). The environmental conditions for the silkworms were maintaining a temperature of 25 °C, a relative humidity of 55%, and a photoperiod consisting of 12 h of light followed by 12 h of darkness.

All silkworms were divided into three groups (control, 50 µM treated (F_50 µM), and 200 µM treated (F_200 µM)). Each group consisted of three replicates, with a total of 50 silkworms in each replicate. For the F_50 µM and F_200 µM groups, the silkworms were fed with mulberry leaves that had been soaked with sodium selenite solution at concentrations of 50 µM and 200 µM from the 1st day of the 4th instar, respectively, while the control group received untreated mulberry leaves. The mulberry leaves were subsequently immersed in the sodium selenite solution for 30 min and then were shade-dried [19]. On the 3rd day of the 5th instar, the fat bodies from all silkworms were excised and placed into RNAase-free collection tubes, with each tube containing four fat bodies. Three replicates per group were utilized for subsequent sequencing analyses [15].

### 4.2. miRNA Sequencing

The sequencing of miRNAs was conducted by the Hangzhou LianChuan Biological Company (Hangzhou, China). In summary, miRNA sequencing libraries were prepared in accordance with the protocols outlined in the TruSeq Small RNA Sample Preparation Kits (Illumina, San Diego, CA, USA). The sequencing process utilized a high-throughput sequencer, specifically the Illumina Hiseq2000/2500, which provided a read length of 1 × 50 base pairs. Low-quality reads were systematically filtered out, and the remaining sequences were subsequently analyzed and compared using the Rfam and Repbase databases. This process yielded validated data that facilitated further analysis of miRNA expression. Differentially expressed miRNAs were identified based on the criteria of log2|fold change| > 1 and *p* < 0.05.

### 4.3. Validation of miRNA by RT-qPCR Analysis

RT-qPCR analysis was employed to validate the 19 differentially expressed miRNAs (DE-miRNAs) across the two treatment groups. The design of primers for differentially expressed miRNAs was initially conducted using the miRprimer software (https://sourceforge.net/projects/mirprimer/, accessed on 14 July 2024), as detailed in Table S5. The detection of differentially expressed miRNAs was performed utilizing the miRNA fluorescence quantitative PCR kit (dye-based method) provided by Sangon Biotech (Shanghai, China) Co. The analysis of the DE-miRNAs was carried out using a fluorescent and quantitative approach, specifically through the application of the SYBR Green I dye method. Each assay was conducted in triplicate for all samples and the resulting data were analyzed using the 2^−ΔΔCt^ method [60].

### 4.4. Target Gene Prediction

The prediction of target genes for significantly differentially expressed microRNAs (miRNAs) was conducted utilizing two distinct software programs: TargetScan (accessed on 16 July 2024) [61] and miRanda [62]. The target genes identified by each software were subsequently filtered based on their respective scoring criteria. Specifically, the TargetScan algorithm excluded target genes with a context score percentile below 50 while the miRanda algorithm eliminated those with a maximum free energy (Max Energy) exceeding −10. Ultimately, the intersection of the target genes predicted by both software programs was considered the definitive set of target genes for the differentially expressed miRNAs (DE-miRNAs) and was employed for further analysis.

### 4.5. Differential miRNA-Target Gene Enrichment Analysis

All target genes corresponding to the selected miRNAs were first imported into the Cytoscape v3.8.0 platform and GO enrichment and KEGG pathway visualization analysis were performed by the ClueGO v2.5.7 plug-in included in the platform [63]. The analysis involved counting the number of genes annotated for each function or pathway. A hypergeometric test was then employed to compare the number of target gene mRNAs associated with the selected miRNAs against the total number of genes linked to the respective GO or KEGG pathways in the annotated libraries, which encompass all genes with functional annotations or all miRNA-target genes with functional annotations. Additionally, *p* ≤ 0.01 was established and functions that satisfied this criterion were classified as significantly enriched within the miRNA–mRNA pairs. This functional significance enrichment analysis serves to elucidate the primary biological functions associated with miRNA–mRNA interactions.

### 4.6. Protein Interaction Network Construction and Hub Gene Screening

The STRING databank can provide us with a comprehensive and objective protein–protein interaction (PPI) network, through which protein–protein interactions can be predicted. In this experiment, target genes significantly enriched in the KEGG pathway were imported into the STRING database and filtered for PPI node pairs with scores greater than 0.9. The results were imported into the cytoHubba plugin in the CellScape platform (https://apps.cytoscape.org/apps/cytohubba, accessed on 18 July 2024), and the node scores were calculated by topology algorithms (Closeness Centrality) to identify the pivotal genes [64].

### 4.7. Construction of the KEGG-miRNA-Target Network

We constructed and visualized a miRNA-target interaction network utilizing Cytoscape. The network topology was analyzed using CytoNCA version 2.1.6 and the significantly differentially expressed miRNAs (DE-miRNAs) were incorporated into the network using the aforementioned algorithm [65].

## 5. Conclusions

Our study demonstrates that Se supplementation has concentration-dependent effects on silkworm development mediated through distinct miRNA regulatory mechanisms. Low concentrations of Se enhance development by fine-tuning immune and metabolic pathways via bmo-miR-2a-1-5p while high concentrations inhibit development by disrupting RNA processing through bmo-miR-317-3p_L-2R+2. Se supplementation displayed dual effects on silkworm development. In total, 50 µM of Se supplement was a benefit to silkworms; however, 200 µM of Se treatment displayed toxic effects. Compared to the control, 84 miRNAs displayed different expression levels in the F_50 µM group with 72 miRNAs up-regulated and 12 down-regulated; 152 miRNAs were differentially expressed in the F_200 µM group, with 124 increased and 28 decreased. In the F_50 µM group, the target genes of differentially expressed miRNA were mainly enriched in the Toll and Imd signaling pathways, oxidative phosphorylation, and ribosome biogenesis in eukaryotes; mainly, this occurred in oxidative phosphorylation, ribosome biogenesis in eukaryotes, and the spliceosome in the F_200 µM group. Additionally, 50 µM of Se improves silkworm development via the bmo-miR-2a-1-5p regulation of 10014128 (DREDD), 100862750 (ATF2), and 101744000 (Tak1) genes, which were enriched in the Toll and Imd signaling pathways. In total, 200 µM of Se inhibits the silkworm through the bmo-miR-317-3p_L-2R+2 targeting 101738508 (SF3b) and 101746688 (Prp19) genes, which were in the spliceosome pathway. Thus, our results demonstrated that Se supplementation improved the silkworm development via the bmo-miR-2a-1-5p miRNA regulation of the Toll and Imd signaling pathways and played an inhibitory role via bmo-miR-317-3p_L-2R+2 miRNA targeting the spliceosome pathway. Our findings bridge nutritional effect and post-transcriptional regulation, offering valuable insights for sustainable insect farming practices.

## Data Availability

All data are contained within the article or its Supplementary Materials as figures or tables.

## Supplementary Materials

The following supporting information can be downloaded at: www.mdpi.com/article/10.3390/ijms26073394/s1.
