# Supplementary material for: MiRNA Omics Reveal the Mechanisms of the Dual Effects of Selenium Supplementation on the Development of the Silkworm (Bombyx mori)
Source: Int J Mol Sci. 2025 Apr 4;26(7):3394. doi: 10.3390/ijms26073394 (PMC11989355; doi:10.3390/ijms26073394)
Supplement: Supplementary file 1 [file ijms-26-03394-s001.zip › ijms-3474843-supplementary.pdf]

Supporting Information

Supporting Figures and Tables

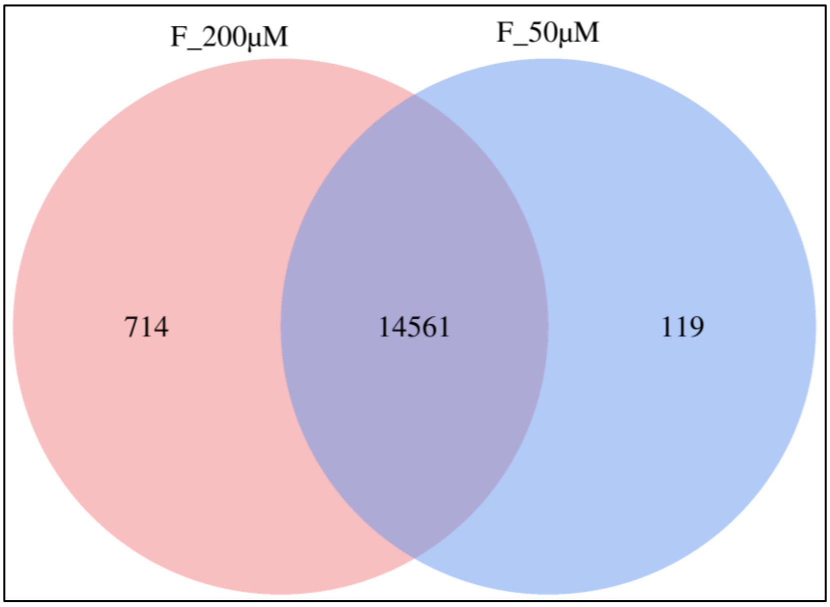

Figure S1. A Venn diagram illustrating the intersection of predicted target gene sites within the F_50μM and F_200μM groups.

Table S1. Statistical analysis of miRNA libraries.

Sample	Raw Reads	Clean Reads	Total Reads	miRNA	Precursor
Control_1	14 643 904	7 255 343	4 967 200	297	248
Control_2	14 091 845	6 674 099	5 308 203	266	229
Control_3	12 961 376	6 493 359	4 431 247	323	263
F1_50µM	10 934 638	4 078 960	4 886 259	411	344
F2_50µM	20 444 542	8 120 667	9 095 435	406	323
F3_50µM	16 527 552	5 430 668	7 391 047	390	390
F4_200µM	13 210 432	3 4177 30	7 884 280	396	322
F5_200µM	14 398 577	5 636 922	6 253 329	423	341
F6_200µM	15 092 331	5 150 945	6 152 181	470	380

Table S2. The most significantly enriched GO terms.

Group	Category	ID	Description	Term *P*
F_50μM	BP-associated category	0006399	tRNA metabolic processes	1.2246×10^{-18}
		0034660	ncRNA metabolic processes	1.7859×10^{-12}
		0009653	anatomical structure morphogenesis	6.6690×10^{-9}
		0048646	anatomical structure formation	1.2153×10^{-8}
		0048468	cell development	2.7808×10^{-8}
	MF-associated category	0003774	molecular activity	4.1918×10^{-10}
		0022836	Gated channel activity	$8.\ 4085 \times 10^{-9}$
		0003676	nucleic acid binding	1.0116×10^{-8}
		0022838	substrate-specific channel activity	2.9183×10^{-8}
		0022803	passive transmembrane transporter activity	2.9183×10^{-8}
	CC-associated category	0044444	cytoplasmic fraction	6.3699×10^{-18}
		0005737	cytoplasmic	1.1725×10^{-15}
		0030529	ribonucleoprotein complex	3.8518×10^{-12}
		0005622	intracellular	7.9423×10^{-9}
		0005840	ribosomes	2.6266×10^{-8}
F_200μM	BP-associated category	0006399	tRNA metabolic process	6.4800×10^{-21}
		0034660	ncRNA metabolic process	6.0400×10^{-14}
		0007018	microtubule-based movement	1.1900×10^{-9}
		0006928	movement of cell or subcellular component	3.3000×10^{-9}
		0051336	regulation of hydrolase activity	3.8600×10^{-7}
	MF-associated category	0003774	motor activity	3.5100×10^{-13}
		0003777	microtubule motor activity	5.0400×10^{-10}
		0022838	substrate- specific channel activity	1.8800×10^{-8}
		0022803	passive transmembrane transporter activity	1.8800×10^{-8}
		0015267	channel activity	1.8800×10^{-8}
	CC-associated category	0044444	cytoplasmic part	9.0600×10^{-21}
		0005737	cytoplasm	1.1725×10^{-15}
		0030529	ribonucleoprotein complex	2.1700×10^{-16}
		0005840	ribosome	4.0900×10^{-15}
		0005739	mitochondrion	2.0500×10^{-9}

Table S3. KEGG pathway enrichment analysis table for target genes.

Group	KEGG pathway	Name	pVal
F_50 μM	KEGG:04624	Toll and Imd signaling pathway	6.0716×10^{-6}
	KEGG:00190	Oxidative phosphorylation	2.2650×10^{-5}
	KEGG:03008	Ribosome biogenesis in eukaryotes	2.4875×10^{-4}
F_200 μM	KEGG:00190	Oxidative phosphorylation	1.4600×10^{-4}
	KEGG:03008	Ribosome biogenesis in eukaryotes	2.7358×10^{-4}
	KEGG:03040	Spliceosome	3.1303×10^{-4}

Table S4. Top 15 nodes in the PPI network.

Group	Name	Closeness	Group	Name	Closeness
F_50 μM	101744000	8.17	F_200 μM	100101178	50.50
	732928	7.25		101744663	50.00
	101744017	7.08		100036587	49.50
	100862753	6.67		732941	49.50
	100126162	6.48		732940	49.00
	100529214	6.35		101736447	48.33
	101736835	6.15		778458	46.50
	692545	6.07		692785	46.50
	100862750	5.73		101738508	45.00
	101743376	5.68		733079	44.83
	101743924	5.42		101738436	44.83
	101743155	5.35		101742628	44.33
	101738264	5.18		100033384	43.83
	101743223	4.93		101746688	43.33
	100141428	4.52		692813	42.67

Table S5. miRNA primers for RT-qPCR.

miRNA	Sequence
bmo-mir-2807d-p3	CGCGCGAACGTTAAAAAACT
tca-miR-283-5p_R+3	CGCGAAATATCAGCTGGTAATT
bmo-miR-1a-3p	GCGCGTGGAATGTAAAGAAGT
bmo-miR-3219	GCGCGGTGAGACTAATATATCCA
bmo-miR-283-5p_L-1R+3	GCGCGAAATATCAGCTGGTAAT
bmo-mir-2744-p5_1ss2AU	GCGCTGCCTTGTCATCG
bmo-mir-6497-p5	CGTGTCGGGTTTGGACG
PC-5p-58410_109	GCGAACGGGCTTGGTAGAA
pxy-mir-6497-p3_1ss10CU	CGCCGCGGATCTTCCTA
bmo-miR-2758-5p	CGCGACTTGGTAGAACACGT
bmo-miR-10-5p_R+1	GCGACCCTGTAGATCCGAA
bmo-miR-277-3p	CGCGTAAATGCACTATCTGGT
dme-miR-10-5p_L+1_1ss23UA	CGCGTACCCTGTAGATCCGAA
bmo-miR-317-3p_L-2R+2	GCGTGAACACAGCTGGTGG
bmo-miR-9c-5p_R+4	GCGCGTCTTTGGTATCCTAGC
bmo-miR-2805_L+4R-1	CGGATGTTCCTGACGAACC
bmo-mir-6497-p3	CGCGCTGGTCGATGTTC
pxy-mir-6497-p5_1ss9CU	GCGCCGTAAGGTCGTGTC
pxy-mir-6497-p5_1ss8CU_3	GCGCGTAAGGTCGTGTCG
bmo-mir-2807d-p3	CGCGCGAACGTTAAAAAACT
Uca-miR-283-5p_R+3	CGCGAAATATCAGCTGGTAATT
bmo-miR-1a-3p	GCGCGTGGAATGTAAAGAAGT
bmo-miR-3219	GCGCGGTGAGACTAATATATCCA
bmo-miR-283-5p_L-1R+3	GCGCGAAATATCAGCTGGTAAT
bmo-mir-2744-p5_1ss2AU	GCGCTGCCTTGTCATCG
